# Genetic integrity of four species of *Leptidea* (Pieridae, Lepidoptera) as sampled in sympatry in West Siberia

**DOI:** 10.3897/CompCytogen.v9i3.4636

**Published:** 2015-06-23

**Authors:** Vladimir I. Solovyev, Yury Ilinsky, Oleg E. Kosterin

**Affiliations:** 1 Institute of Cytology & Genetics of Siberian Branch of Russian Academy of Sciences, Acad. Lavrentyev ave. 10, Novosibirsk, 630090, Russia; 2 Novosibirsk State University, Pirogova str. 2, Novosibirsk, 630090, Russia; 3 Institute of Chemistry and Biology, Immanuil Kant Baltic Federal University, Alexander Nevsky str. 14, 236038 Kaliningrad, Russia

**Keywords:** *Leptidea*, Lepidoptera, *Wolbachia*, introgression, molecular markers, histone H1, *COI*, *ITS2*, *wsp*, genitalia morphology, intraspecific variation

## Abstract

In southern West Siberia, as many as four *Leptidea* Billberg, 1820 species are present sympatrically: *Leptidea amurensis* (Ménétriés, 1859), *Leptidea morsei* (Ménétriés, 1859), *Leptidea sinapis* (Linnaeus, 1758) and *Leptidea juvernica* Williams, 1946. The two latter were recently recognised as nearly sibling species on morphological and molecular characters. Specimens intermediate as to their subtle diagnostic characters occurring in West Siberia and elsewhere were interpreted as resulted from limited introgression. This supposition was tested via populational morphological and molecular analysis of spring brood specimens of all the four species taken from a limited (4.5 × 0.2 km) area in the suburbs of Novosibirsk. The samples were analysed with respect to the genitalic morphology, external characters, three nuclear (*CAD*, *H1* gene and *ITS2*) and one mitochondrial (*COI*) molecular markers, infection of the intracellular maternally inherited bacterial symbiont *Wolbachia* Hertig, 1836 and its *wsp* gene coding for a hypervariable surface protein. Interspecific variation of the nuclear *CAD* and *ITS2* sequences and the mitochondrial *COI* gene in *Leptidea sinapis* and *Leptidea juvernica* turned out concordant. The absence of molecular evidence of introgression suggests genetic integrity of these two species and allows their reliable identification by molecular characters. The genitalic (lengths of the saccus and valva) and external characters (wing pattern) of males overlap in *Leptidea sinapis* and *Leptidea juvernica*, as identified by molecular markers and thus are not so helpful in actual species identification. Only the ductus bursae length showed no overlap and can be used for identification of females. The histone H1 gene appeared five times less variable over the four studied species than *COI*, and found to be identical in species *Leptidea sinapis* and *Leptidea juvernica*. *Wolbachia* infection was found in all studied species. We identified three *wsp* variants of *Wolbachia*: 1) *wsp-10* allele in *Leptidea amurensis*, *Leptidea sinapis*, *Leptidea juvernica*; 2) a very similar *wsp-687* allele in *Leptidea sinapis*; and 3) *wsp-688*, highly divergent to the previous ones, in *Leptidea morsei*.

## Introduction

The genus *Leptidea* Billberg, 1820 (Dismorphiinae, Pieridae) includes several (at least eight) Palearctic species. Recently it attracted attention because of repeated and rather unexpected discoveries of sibling species (Dincă et al. 2011). Firstly, *Leptidea lorkovicii* Réal, 1988 was separated from the sympatric *Leptidea sinapis* (Linnaeus, 1758) on the basis of substantial differences in the genitalia structure (Réal 1988). Later a new name *Leptidea reali* Reissinger, (1990) was proposed to it because of existence of a senior homonym *Leptidea duponcheli lorkovici* Pfeiffer, 1932 (Reissinger 1990). Note that later it was found out (Dincă et al. 2011) that the new name was not necessary and invalid because of existence of an older available name *juvernica* Williams, 1946 proposed for an Irish population showing the relevant morphology. Secondly, on the basis of molecular and karyological data, the species known under the invalid name *Leptidea reali* was split into two allopatric species, *Leptidea reali* s. str. from Spain, southern France and Italy (for this species the name in narrow sense is valid) and *Leptidea juvernica* Williams, 1946 ranging from the French Pyrenees in the south-west and Ireland in the north-west to Central Siberia in the east (Dincă et al. 2011). Citing literature data *de facto* dealing with *Leptidea juvernica*, below we will use this name although before 2011 the authors used the name *Leptidea reali*. Ranges of both *Leptidea reali* and *Leptidea juvernica* overlap with that of *Leptidea sinapis* ranging from Spain and Ireland to East Siberia (Dincă et al. 2011). Hence *Leptidea sinapis* and *Leptidea juvernica* co-occur on a vast territory from South Europe to Central Siberia.

The main diagnostic character of *Leptidea reali* and *Leptidea juvernica* versus *Leptidea sinapis* is a substantially greater relative lengths of the aedeagus and saccus (which correlate to each other) in the male genitalia and of the ductus bursae in the female genitalia (Fumi 2008, Ivonin et al. 2009, Dincă et al. 2011, Sachanowicz 2013). There are less distinct differences between *Leptidea juvernica* and *Leptidea sinapis* in the wing shape and pattern. For both the European part of Russia (Bolshakov 2005, Bolshakov et al. 2013) and West Siberia (Ivonin et al. 2009), the following differences between *Leptidea juvernica* and *Leptidea sinapis* in the wing coloration were claimed:

- spring brood males of *Leptidea juvernica* have in general darker, more suffused hind wing underside below vein M3 and with less distinct stripy pattern (less expressed lighter postdiscal spots between veins) than those of *Leptidea sinapis*;
- summer brood males of *Leptidea juvernica* differ from those of *Leptidea sinapis* in the fore wing upperside without the light rim along the apical dark spot and darkened ends of veins M3 and Cu1;
- in West Siberia, the spring brood males of *Leptidea juvernica* were claimed to have a more attenuated fore wing apex than those of *Leptidea sinapis* (Ivonin et al. 2009).

No external differences were revealed between females of the two species.

Authors working in different regions (Verovnik and Glogovčan 2007, Ivonin et al. 2009, Bolshakov et al. 2013) pointed out a substantial variation in these two species. Several morphs were recognised based on the tint of wing coloration, which are shared by both *Leptidea sinapis* and *Leptidea juvernica* (Bolshakov et al. 2013). The lengths of the aedeagus and saccus taken alone do not allow distinguishing *Leptidea sinapis* from *Leptidea juvernica* in all cases because of some overlap in *Leptidea sinapis* and *Leptidea juvernica* (Fumi 2008, Sachanowicz 2013). Tsvetkov (2007) reported that samples of *Leptidea juvernica* from forested and open habitats differed in the average relative aedeagus length, which is less in forested habitats. It was even hypothesized that such variation could be supported by selection for a longer aedeagus to ensure mating in more windy open habitats (Bolshakov et al. 2013); however, this would demand a long lasting maintenance of genetic isolation between the two habitat types, which hardly exists at all. Bolshakov et al. (2013) reported specimens with intermediate genitalia from Mordovia Republic (European Russia), e.g. males of *Leptidea juvernica* with a normally long aedeagus but the saccus short as in *sinapis* and curved as in *juvernica*. Ivonin et al. (2009) reported the occurrence in Novosibirsk Province (West Siberia, Russia) of external characters of *Leptidea juvernica* among males with the genitalia of *Leptidea sinapis* (with a short and straight saccus) but not vice versa, for no external characters of *Leptidea sinapis* were found in males with the genitalia of *Leptidea juvernica* (with a long and S-like curved saccus). Verovnik and Glogovčan (2007) reported the occurrence of males that were intermediate between *Leptidea sinapis* and *Leptidea juvernica* in Slovenia, namely: (i) with the aedeagus of intermediate length; (ii) with long saccus but short aedeagus and (iii) with the genitalia of *Leptidea juvernica* but closer to *Leptidea sinapis* according to RAPD markers.

These facts can be interpreted in three ways: (i) as resulting from some gene exchange (introgression) between *Leptidea sinapis* and *Leptidea juvernica*; (ii) as common polymorphism of genes affecting the genitalia structure and/or wing coloration, inherited from the common ancestor, or (iii) by independent mutations (homoplasy) of these hypothetical genes.

The relationships of closely related species may be clarified via two approaches, the phylogeographic and population genetic ones. The former approach implies accumulation of data from a territory as broad as possible in order to reconstruct the history of divergence and spread of species. The latter approach consists of analysing large samples from certain populations in order to register phenomena such as deviations from panmixia, linkage disequilibrium, gene exchange between sympatric taxa, and effects of natural selection.

Relationships between sibling species of *Leptidea* were mostly studied via the phylogeographic approach applied to the entire species ranges (Lukhtanov et al. 2011, Dincă et al. 2011, 2013) using the mitochondrial *COI* and *ND1* genes, the nuclear *CAD*, *ITS2*, and *Wg* markers, and karyotype. These studies did not reveal any introgression between *Leptidea reali*, *Leptidea juvernica* and *Leptidea sinapis* (Lukhtanov et al. 2011, Dincă et al. 2011, 2013). Examples of population genetic studies are the analysis of several sympatric populations of *Leptidea reali* and *Leptidea sinapis* in the French Pyrenees (Martin et al. 2003) and of *Leptidea juvernica* and *Leptidea sinapis* in Slovenia (Verovnik and Glogovčan 2007). Martin et al. (2003) rejected the introgression hypothesis while Verovnik and Glogovčan (2007) did not exclude some degree of gene exchange between *Leptidea sinapis* and *Leptidea juvernica*.

Another approach is searching for particular mechanisms of isolation between these two species. Hybridisation experiments revealed that prezygotic isolation between *Leptidea sinapis* and *Leptidea juvernica* or *Leptidea reali* was probably based on behavioral barriers, for instance recognition by females of a species-specific courtship behaviour of males or species-specific pheromones (Friberg et al. 2008b, Dincă et al. 2013). Non-conspecific matings between these species were not observed while conspecific individuals from geographically remote populations mated successfully (Dincă et al. 2013). A shifted flight period and some habitat segregation also contribute to the prezygotic isolation between *Leptidea sinapis* and *Leptidea juvernica* (Friberg et al. 2008a). Differences in larval foodplant species were not found (Friberg and Wiklund 2009).

**Figure 1.**
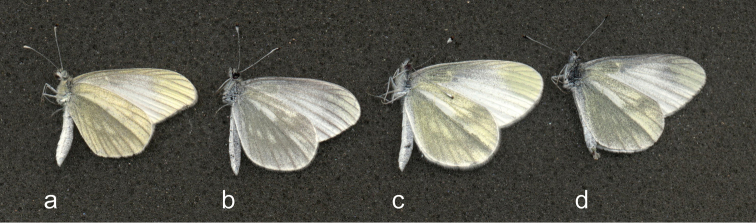
Spring brood males of four species of *Leptidea* Billberg, 1820: *Leptidea amurensis* (Ménétriés, 1859) (**a**), *Leptidea morsei* (Fenton, 1881) (**b**), *Leptidea sinapis* (Linnaeus, 1758) (**c**) and *Leptidea juvernica* Williams, 1946 (**d**), simultaneously collected in the studied area at the border of Novosibirsk city and Berdsk town, West Siberia, Russia (after Ivonin et al. 2009). Note the difference in the shape of the fore wing apex.

The western foothills of the Altay-Sayan Mountain System (West Siberia, Russia) are unique in being inhabited by four *Leptidea* species altogether, more than elsewhere in the world: *Leptidea morsei* (Fenton, 1881), *Leptidea amurensis* (Ménétriés, 1859), *Leptidea sinapis* and *Leptidea juvernica* (Fig. 1). They have different habitat preferences: at least in Novosibirsk Province: *Leptidea morsei* mostly inhabits open woods, *Leptidea sinapis* — various meadows, *Leptidea amurensis* and *Leptidea juvernica* mostly inhabit meadow steppes in rough relief terrains (Ivonin et al. 2009). In spite of these preferences, all the four species coexist with nearly equal abundance and similar flight period on grassy glades on the eastern bank of the Novosibirsk Water Reserve in the Novosibirsk Academy Town (Kosterin et al. 2007), making this territory an excellent site for studying isolation *vs* introgression of *Leptidea* spp. Hence, we attempted a pure population genetic approach and analysed a sample of spring brood specimens of *Leptidea* collected from the same small area at the junction of the Novosibirsk city and Berdsk town.

The main attention was paid to the closely related and supposedly hybridising species *Leptidea sinapis* and *Leptidea juvernica*. They were analysed with respect to the popular mitochondrial marker *COI* (the gene for cytochrome oxidase I) and the nuclear markers *CAD* (the gene for carbamoyl phosphate synthetase II, aspartate carbamoyltransferase, dihydroorotase), *ITS2* (internal transcribed spacer 2 in the ribosome cluster), and the histone H1 gene, designated here as *H1*. A histone H1 gene was recently proposed as a good phylogenetic marker (Zaytseva et al. 2012, 2015, Solovyev et al. 2015). We also analysed infection by the maternally inherited endosymbiont *Wolbachia* Hertig, 1836 (Zhou et al. 1998; Van Meer et al. 1999; Baldo and Werren 2007), and sequences of its highly variable gene *wsp* (*Wolbachia* surface protein). In addition, the males of *Leptidea sinapis* and *Leptidea juvernica* were analysed for the lengths of the valve and saccus in the male genitalia and scored for the wing characters, and the ductus bursae was measured in females. The *COI*, histone *H1* and *wsp* genes were also sequenced and the *Wolbachia* infection was assessed in the two other co-occurring species, *Leptidea morsei* and *Leptidea amurensis*. Since their external characters are constant and sufficient for reliable identification, their genitalia were not examined.

## Materials and methods

### Material

**Figure 2.**
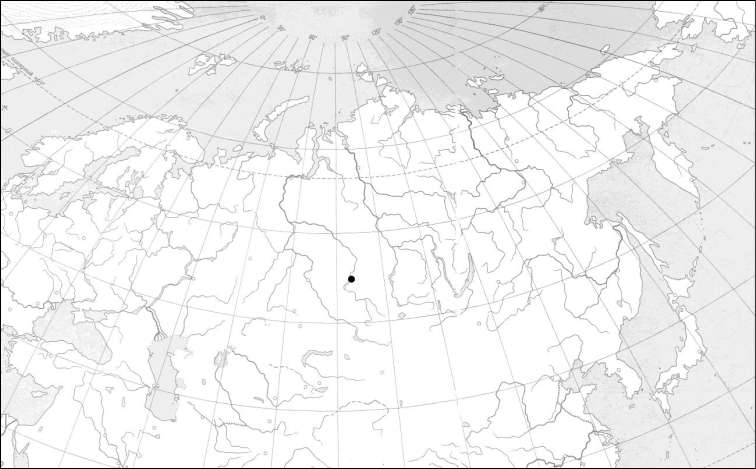
Position (black circle) of the studied area at the border of Novosibirsk city and Berdsk town (54°47'37"N; 83°04'34"E – 54°50'04"N; 83°04'40"E; DMS), Novosibirsk Province, Russia, on a schematic map of northern Eurasia.

Seventy spring brood specimens of *Leptidea* spp. were collected in the vicinity of Novosibirsk Academy Town, Novosibirsk Province. The collection area was a 100–200 m wide and a 4.5 km long continuous stripe of meadows adjacent to birch/ pine forests, along the bank of the Novosibirsk Water Reserve and the parallel railroad, between Obskoe More railway station (54°47'37"N; 83°04'34"E; (DMS)) and a point (54°50'04"N; 83°04'40"E (DMS)), 900 m NNE of Rechkunovka railway station, at elevations of 107–137 m a.s.l. (see the locality on a schematic map of northern Eurasia in Fig. 2). Note that the northern half of this area belongs to the city of Novosibirsk while the southern half to the satellite town of Berdsk (with the border at Beregovaya railway station in the middle of the collecting area). Berdsk is the type locality of the subspecies *Leptidea reali yakovlevi* Mazel, 2003 (the justification of which to our opinion have been insufficiently reasoned in the original description). The specimens were collected by O.E. Kosterin in June 2010, May 2011 and May 2012 with a net and frozen immediately. Details of the specimens examined are provided in Table 1. In screening for *Wolbachia* infection, the combined sample of *Leptidea juvernica* and *Leptidea sinapis* was updated with 15 more specimens which were not identified to either of these two species, not analysed in other respects and not included into Table 1, so that the total sample of *Wolbachia* screening contained 85 *Leptidea* specimens.

**Table 1.** Material collected, *COI* gene allelic states as revealed by CAPS approach (denoted as follows: s – *Leptidea sinapis*, j – *Leptidea juvernica*, a – *Leptidea amurensis*, m – *Leptidea morsei*), European Nucleotide Archive (ENA) accession numbers of the *COI* and *H1* gene sequences, presence of *Wolbachia* infection (+ detected; - not detected) and the *wsp* alleles according to the PubMLST database.

Specimen	Sex	Date collection	*COI* variant	*COI* ENA accession number	*H1* ENA accession number	*Wolbachia* infection (*wsp* allele)
L1	♂	05.06.2010	s			-
L2	♂	05.06.2010	j			+
L3	♂	05.06.2010	j			+
L4	♂	05.06.2010	s			+
L5	♂	05.06.2010	j			+
L6	♂	05.06.2010	s			+
L7	♂	06.06.2010	s			+
L8	♂	06.06.2010	j			+
L9	♂	06.06.2010	j			+
L10	♂	05.06.2010	j	HG969218	LN606440	+
L11	♂	29.05.2011	j	HG969219		+
L12	♂	29.05.2011	j	HG969220	LN606441	+ (*wsp*-10)
L13	♂	29.05.2011	j	HG969221		+
L14	♂	29.05.2011	j	HG969222		+
L15	♂	29.05.2011	s	HG969223	LN606442	+
L16	♂	29.05.2011	s	HG969224	LN606443	+ (*wsp*-687)
L17	♂	29.05.2011	s	HG969225	LN606444	+ (*wsp*-10)
L18	♂	29.05.2011	s	HG969226		+
L19	♂	13.05.2012	j	HG969227		+
L20	♂	13.05.2012	j			+
L21	♂	13.05.2012	j			+
L22	♂	13.05.2012	s			+
L23	♂	13.05.2012	s			+
L24	♂	14.05.2012	j			+
L25	♂	14.05.2012	s			+
L26	♂	15.05.2012	s			-
L27	♂	15.05.2012	j			+
L28	♂	15.05.2012	j			+
L29	♀	29.05.2010	j			+
L30	♀	05.06.2010	j			+
L31	♀	05.06.2010	j			+
L32	♀	05.06.2010	j			+
L33	♀	05.06.2010	j			+
L34	♀	05.06.2010	s			-
L35	♀	05.06.2010	j			+
L36	♀	06.06.2010	j			+
L37	♀	06.06.2010	s			+
L38	♀	06.06.2010	j			+
L39	♀	13.05.2012	j			+
L40	♀	13.05.2012	j			+
L41	♀	13.05.2012	j			+
L42	♀	13.05.2012	j			+
L43	♀	14.05.2012	s			+
L44	♀	14.05.2012	s			+
L45	♀	14.05.2012	j			+
L46	♀	14.05.2012	s			+
L47	♂	29.05.2010	a	HG969228		+
L48	♂	29.05.2010	a	HG969229		+
L49	♂	26.05.2011	a	HG969230		+
L50	♂	26.05.2011	a	HG969231	LN606445	+
L51	♂	29.05.2011	a	HG969232		+
L52	♂	29.05.2011	a	HG969233		+
L53	♂	29.05.2011	a	HG969234		+
L54	♀	28.05.2010	a	HG969235		+
L55	♀	28.05.2010	a	HG969236		+
L56	♀	26.05.2011	a	HG969237		+
L57	♀	26.05.2011	a	HG969238		+
L58	♀	26.05.2011	a	HG969239	LN606446	+ (*wsp*-10)
L59	♀	29.05.2011	a	HG969240		+
L60	♂	29.05.2010	m	HG969241	LN606447	+
L61	♂	29.05.2011	m	HG969242		+
L62	♂	29.05.2011	m	HG969243		+
L63	♂	29.05.2011	m	HG969244		+
L64	♂	29.05.2011	m	HG969245		+
L65	♂	29.05.2011	m	HG969246		+
L66	♀	29.05.2010	m	HG969247		+
L67	♀	26.05.2011	m	HG969248	LN606448	+ (*wsp*-686)
L68	♀	29.05.2011	m	HG969249		+
L69	♀	29.05.2011	m	HG969250		+
L70	♀	29.05.2011	m	HG969251		+

### DNA extraction

Genomic DNA was extracted according to Bogdanova et al. (2009), with modifications for isolation from individual insects. Frozen specimens without abdomen and wings were homogenized in 0.6 ml 0.15 M NaCl. The homogenate was centrifuged (3,300 rcf, 5 min) and the supernatant was discarded, then 0.2 ml solution for DNA extraction (0.1 Tris-HCl, pH 8.0; 5 mM EDTA; 0.5% SDS; 0.1 M NaCl) was added and incubated at room temperature for 40 minutes. Then the solution was centrifuged (16,100 rcf, 5 min) and the pellet was discarded. To remove proteins and RNA, LiCl (0.2 ml, 5M) was added to the supernatant solution and incubated on ice for 15 min. The solution was centrifuged (16,100 rcf, 5 min) and the supernatant was transferred to fresh tubes. Ethanol (1 ml, 96%) was added and the mixture was incubated on ice for an hour. Then it was centrifuged (16,100 rcf, 10 min) and the supernatant was discarded. The precipitate was washed with 0.1 ml 75% ethanol and centrifuged (16,100 rcf, 5 min), then dried at 50 °C for 5 min and dissolved in 50 μl of deionized H_2_O.

### DNA amplification and sequencing

A 708 bp long fragment of the *COI* gene, positions 1526–2156 (positions are given according to the mitochondrial reference of *Drosophila yakuba* Burla, 1954 (AN X03240)), was amplified with the universal insect primers LCO-1490 and HCO-2198 (Folmer et al. 1994). A 684 bp sequence of *ITS2* (internal transcribed spacer 2) and 571 bp sequence of *CAD* (carbamoyl phosphate synthase II, Aspartate carbamoyltransferase, dihydroorotase) were amplified with primer pairs ITS3/ITS4 and CADFa/CADRa, respectively, following Dincă et al. (2011). The *H1* gene was amplified with primers designed for two overlapping sequences: the 5’ terminal part with the primer pair LH4-f (5’ACCCTGTACGGTTTCGGCGGTTAA) and HeH1-r (5’ AGCGCCCTTGCCCTTGGTCTGTATC) and the 3’terminal part of gene with another pair, HeH1-f (5’ACCCACCCCAAGACCTCCGAGATGGT) and LeH1C-r (5’AGGGGGACTCACTTTTTGGA). The 5’ terminal fragment is approximately 1.5 kbp long, and the 3’-terminal fragment is 650 bp long. The primers were originally designed to match orthologous sequences of *Bombyx mori* (Linnaeus, 1758) (LH4-f), *Heliconius erato* (Linnaeus, 1758) (HeH1-r and HeH1-f) (Solovyev et al. 2015) and *Leptidea sinapis* (LeH1C-r); they were produced by Biosset (Novosibirsk. Russia).

DNA samples were examined for *Wolbachia* infection by amplification of *wsp* with the following primer set: wsp81F (5’TGGTCCAATAAGTGATGAAGAAAC-3’), wsp691R (5’AAAAATTAAACGCTACTCCA-3’) (Braig et al. 1998). PCR products of five DNA stocks of four species were sequenced.

PCR mixtures (30 μl) contained 0.2 mM of each dNTP, 1.5 mM MgCl_2_, 25mM KCl, 60 mM Tris-HCl (pH 8.5), 10 mM β-mercaptoethanol, 0.1% Triton X-100, 0.5 µM of each primer, 1 μl of genomic DNA solution and 1 U of Taq DNA polymerase or 1 U of Smart-Taq DNA Polymerase (by Laboratory Medigen, Novosibirsk, Russia). PCR was performed using a thermal cycler MyCycler (Bio-Rad, USA) with the following program: 1) 94 °C — 2 min 30 s, 1 cycle; 2) 95 °C — 15 s, 47–55 °C — 30 s, 68 °C — 1 min, 35 cycles; 3) 68 °C — 2 min, 1 cycle.

The entire coding sequence of *H1* and a 631 bp long fragment of *COI* (positions 1526–2156) were sequenced. The Sanger reaction was conducted in 30 μl volume of mixture containing 1 μl of BigDye Terminator, version 3.1 (Applied Biosystems), 100–200 ng of DNA, 3 pmol of primer and 6 μl of buffer solution for BigDye 3.1. A MyCycler (Biorad) thermocycler was used with the following program: 95 °C — 45 s, 50 °C — 30 s, 60 °C — 4 min; 26 cycles. Sequencing was made at the SB RAS Genomic Core Facility, Novosibirsk.

Sequence alignments and calculation of the genetic distances were performed using the MEGA 5.0 software package (Tamura et al. 2011).

### *CAPS* genotyping *Leptidea sinapis* and *Leptidea juvernica*

For genotyping the *Leptidea sinapis* and *Leptidea juvernica* specimens with respect to certain diagnostic nucleotide substitutions in mitochondrial and nuclear markers, CAPS analysis was conducted (Konieczny and Ausubel 1993). After the analysis of DNA sequences of *Leptidea sinapis* and *Leptidea juvernica* in public databases, we picked the set of endonucleases *Hpa*II, *Alu*I and *Hind*III for genotyping the *COI* gene, *ITS2* region and *CAD* gene, respectively.

The 708 bp long amplified fragment of *COI* of *Leptidea juvernica* contains three restriction sites for endonuclease *Hpa*II and is digested to 4 fragments (66, 109, 206, 327 bp), while the orthologous fragment of *Leptidea sinapis* has no restriction sites. The *ITS2* region of *Leptidea sinapis* contains the only site specific for endonuclease *Alu*I and is digested into 2 fragments (412, 272 bp); the *ITS2* of *Leptidea juvernica* does not contain restriction sites for *Alu*I. The *CAD* sequence of *Leptidea juvernica* includes only one restriction site for endonuclease *Hind*III, which digests it into two fragments, 110 and 461 bp in length; the *CAD* sequence of *Leptidea sinapis* has two sites which produce three digestion fragments (110, 189, 272 bp). The buffers and enzymes for restriction reactions were produced by Sibenzim, Novosibirsk, Russia. The identical procedure was used for different markers, as follows: 9 μl of the PCR product was added with 0.5 U of endonuclease and 1 μl of a buffer relevant to the endonuclease. The mixture was incubated at 37 °C for 2 hr, inactivated at 80 °C for 20 min and analyzed by electrophoresis in 1.5% agarose.

### Genitalia morphometrics

**Figure 3.**
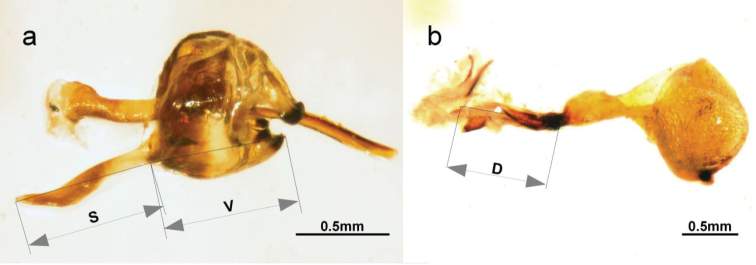
Male (**a** specimen L12) and female (**b** specimen L45) genitalia of *Leptidea juvernica*, with measured parameters indicated, namely the length of the saccus (S), valve (V) and ductus bursae (D).

The abdomen tip with the genitalia was taken from frozen specimens of *Leptidea juvernica* and *Leptidea sinapis*, incubated for 10 min at 98 °C in 10% potassium hydroxide for maceration and dissected under a stereomicroscope. Lengths of the valve (V) and saccus (S) were measured with an ocular-micrometer and binocular lens MBS-2, as shown in Fig. 3. Besides, the saccus curvature was classified as referring to arbitrary binary scores: 0 – straight, 1 – S-like curved.

Statistical analyses were carried out using MS Excel 10 for Windows.

The genitalia were analysed before molecular analysis, which was carried out blindly of the genitalic results. The specimens in which molecular results appeared discordant with morphological ones, were then rechecked for morphology and discordancy was confirmed.

### External characters

**Figure 4.**
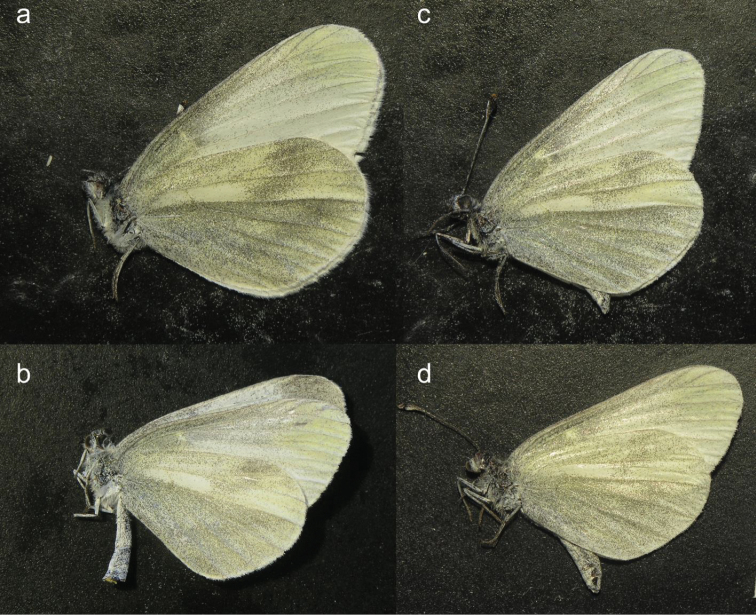
Males of *Leptidea sinapis* (**a** specimen L1 **b** specimen L26) and *Leptidea juvernica*, (**c** specimen L3 **d** specimen L8), with different scores of subjectively evaluated wing characters: the hind wing underside suffusion below vein M3: 0 – lighter, with better expressed lighter spots between veins; 1 – stronger, with scarcely seen lighter spots; and the fore wing apex shape: 1 – more acute, 0 – more rounded. The scores for the shown specimens are as follows (suffusion, apex shape): **a** (0,0); **b** (1,0); **c** (1,1); **d** (1,0); **a** and **c** are variants most frequent in the respective species.

The external characters reported to be different in the spring brood males of *Leptidea juvernica* and *Leptidea sinapis*, namely (1) the wing underside below vein M3 more suffused by dark scales and with less expressed lighter spots between veins and (ii) more attenuate fore wing apex in the former species (Ivonin et al. 2009), are difficult to measure and somewhat subjective. Therefore we classified them as referring to arbitrary binary classes:

- hind wing underside suffusion below vein M3: 0 – with well-expressed lighter spots between veins, 1 – stronger, more even, with scarcely or expressed lighter spots;
- fore wing apex: 0 – broadly rounded; 1 – more attenuated and acute (Fig. 4).

## Results

### Inter- and intraspecies variation of mitochondrial *COI* gene

The 631 bp long fragment of the mitochondrial gene *COI* (position 1526 – 2156) was sequenced for 34 *Leptidea* specimens (10 of *Leptidea sinapis* + *Leptidea juvernica*, 11 of *Leptidea morsei* and 13 of *Leptidea amurensis*) collected in the same locality. The sequences were submitted to European Nucleotide Archive (ENA), for accession numbers see Table 1. The sequences of *Leptidea morsei* specimens were identical. In *Leptidea amurensis*, two alleles were found which differed in position 1969, occupied by either T or A. The 1969T allele was found in 12 specimens while the 1969A allele was only found in only specimen L57. Six *COI* alleles were revealed in *Leptidea sinapis* and *Leptidea juvernica*. These six alleles differed in 22 sites (Table 2) and formed two groups of three alleles each, further referred to as the *s*- and *j*-alleles. The consensuses of each group differed in 17 substitutions, the other 5 substitutions were not diagnostic. The *j*-alleles differed from each other in substitutions in the positions 1615, 1686, 1917. Two of the *s*-alleles differed in T/A substitution in position 2076, while the third, found in specimen L15, has positions 1587 and 1674 occupied by the nucleotides otherwise specific for *j*-alleles. Hence the two latter positions were not diagnostic. As a result, the set of positions diagnostic for the *s*- and *j*-type, which allows species identification, included 15 positions (Table 2).

**Table 2.** Polymorphic positions in the *COI* gene in *Leptidea sinapis* (specimens L15-L18) and *Leptidea juvernica* (specimens L10–L14 and L19). Positions discriminating *s*- and *j*- allele types are boldfaced; intraspecific substitutions are underlined.

Specimens representing six alleles		L10, L11, L14	L12, L13	L19	L15	L16, L17	L18
allele type		**j**	**j**	**j**	**s**	**s**	**s**
Position	**1530**	**T**	**T**	**T**	**C**	**C**	**C**
	1587	A	A	A	A	G	G
	**1599**	**C**	**C**	**C**	**T**	**T**	**T**
	1615	A	G	G	G	G	G
	**1624**	**A**	**A**	**A**	**G**	**G**	**G**
	**1659**	**T**	**T**	**T**	**C**	**C**	**C**
	1674	G	G	G	G	A	A
	1686	T	C	C	C	C	C
	**1720**	**C**	**C**	**C**	**T**	**T**	**T**
	**1854**	**C**	**C**	**C**	**T**	**T**	**T**
	**1860**	**A**	**A**	**A**	**G**	**G**	**G**
	**1914**	**T**	**T**	**T**	**C**	**C**	**C**
	1917	C	C	T	T	T	T
	**1926**	**C**	**C**	**C**	**T**	**T**	**T**
	**1947**	**A**	**A**	**A**	**G**	**G**	**G**
	**1959**	**C**	**C**	**C**	**T**	**T**	**T**
	2076	T	T	T	T	T	A
	**2103**	**C**	**C**	**C**	**T**	**T**	**T**
	**2121**	**T**	**T**	**T**	**A**	**A**	**A**
	**2133**	**G**	**G**	**G**	**A**	**A**	**A**
	**2148**	**C**	**C**	**C**	**T**	**T**	**T**

The averaged and minimum *p*-distances between of the studied *COI* fragment between *Leptidea juvernica* (*j*-alleles), *Leptidea sinapis* (*s*-alleles) *Leptidea morsei*, *Leptidea amurensis*, of are provided in Table 3.

**Table 3.** Evolutionary Divergence over Sequence Pairs between *Leptidea* species in the studied sample as calculated from 34 *COI* gene sequences obtained. The number of nucleotide substitutions per site averaged over all possible specimens pairs for any two species, ± its standard error, is shown below the main diagonal, their minimum value among all specimens pairs for any two species is shown above the main diagonal. The total number of positions was 631.

	1	2	3	4
1. *Leptidea juvernica*		0.024	0.041	0.052
2. *Leptidea sinapis*	0.029±0.006		0.043	0.052
3. *Leptidea morsei*	0.043±0.008	0.043±0.008		0.048
4. *Leptidea amurensis*	0.055±0.008	0.053±0.008	0.048±0.008	

### The *wsp* gene variation

PCR amplification of the *wsp* gene revealed *Wolbachia* infection in 38 of 42 tested males and 18 of 19 tested females of the *Leptidea sinapis* + *Leptidea juvernica* united sample (91.8% prevalence), in all 11 tested specimens of *Leptidea morsei* and in all 13 tested specimens of *Leptidea amurensis* (100% prevalence) (Table 1, but 15 specimens of *Leptidea sinapis* or *Leptidea juvernica*, analysed only for *wsp*, are not included into the table).

The *wsp* gene was sequenced in one specimen of each *Leptidea amurensis* (L58), *Leptidea morsei* (L67) and *Leptidea juvernica* (L12) and two specimens of *Leptidea sinapis* (L16, L17). The sequences were submitted to the PubMLST database http://pubmlst.org [accessed 30 January 2015] (for accession numbers see Table 1). *Leptidea amurensis*, *Leptidea juvernica* and one specimen (L17) of *Leptidea sinapis* turned out to have allele *wsp*-10, with the following hypervariable regions: *HVR1-10*, *HVR2-8*, *HVR3-10*, *HVR4-8*. The specimen L16 of *Leptidea sinapis* had *wsp-687* allele, which differed from *wsp*-10 with one non-synonymous nucleotide substitution A193G (serine to glycine). This allele had not been previously recorded and was designated at http://pubmlst.org as *wsp-687*. *Leptidea morsei* had a *Wolbachia* strain with another new allele, designated as *wsp*-688. This allele differed from *wsp-10* with 81 nucleotide substitutions (uncorrected *p*-distance 0.169) and gaps, resulting in 41 amino acid differences, and had the following hypervariable regions: *HVR1-2*, *HVR2-267*, *HVR3-2*, *HVR4-23*.

### Concordance of mitochondrial and nuclear markers in *Leptidea sinapis* vs *Leptidea juvernica*

The CAPS approach (see ‘Materials and methods’) allowed us to test 36 more specimens of *Leptidea sinapis*/*Leptidea juvernica* in addition to those 10 in which *COI* was sequenced. We distinguished *s*- versus *j*-alleles of the mitochondrial marker *COI* and nuclear markers *CAD* and *ITS2* in the same set of specimens. The three sets of CAPS data, for all three markers, were fully concordant: each specimen possessed either only *s*- or only *j*-alleles for all three markers. This gave us a reason to consider and further refer these specimens as belonging to the true biological species *Leptidea sinapis* and *Leptidea juvernica*, respectively.

The 747 bp long coding sequence of the *H1* gene of histone H1 was sequenced in 9 specimens: L10, L12 (*Leptidea juvernica*), L15–L17 (*Leptidea sinapis*), L50, L58 (*Leptidea amurensis*), L60, L67 (*Leptidea morsei*); the sequences were submitted to ENA (for the accession numbers see Table 1). *Leptidea sinapis* and *Leptidea juvernica* appeared to have identical primary structure of the *H1* coding sequence. Comparison of those of *Leptidea sinapis*, *Leptidea juvernica*, *Leptidea morsei* and *Leptidea amurensis* revealed 10 polymorphic sites, seven of which reside in the region coding for the C-terminal domain. As compared to the consensus *H1* coding sequence for all the four species, the *H1* sequence of *Leptidea morsei* has two transitions, G570A and A654G, while that of both *Leptidea sinapis* and *Leptidea juvernica* has two transitions, G27A and G63A, and two transversions, C456G and A648C. *H1* of *Leptidea amurensis* has three transitions G36A, G346A, G456A, and 1 transversion C453G. The substitution G346A was in the codon first position and lead to the amino acid substitution A116T, while all other above mentioned substitutions are in the third positions and synonymous. Besides, the sequenograms of both studied specimens of *Leptidea amurensis* showed in position 306 overlapping peaks for G (as in the consensus) and T (synonymous substitutions). In one of those specimens (L50), analogous simultaneous presence of both C and T was revealed in position 219. This could result from either heterozygosity for two alleles in homologous histone gene clusters and/or cis-heterogeneity for the repeated *H1* copies in the same cluster.

### Correlation of molecular markers and morphological characters in the group *Leptidea sinapis* + *Leptidea juvernica*

The lengths of the following genital structures were measured: the saccus and valve in males (Tables 4 and 5) and the ductus bursae in females (Tables 4 and 6). Besides, in males, we qualitatively evaluated additional characters such as the shape of saccus (straight versus S-like curved) and some wing characters (Table 7). Females of these species did not differ in external characters.

**Table 4.** The genital measurements of the studied samples of *Leptidea* spp. The mean values and standard deviations are given of the lengths for the saccus (*S*), valve (*V*) and their ratio (*S*/*V*) in the male genitalia and the length of the ductus bursae (*D*) in the female genitalia in the studied samples of *Leptidea sinapis* and *Leptidea juvernica*, as identified by molecular markers, and the united sample of both species.

Parameter	Sample	*S* mm	*V* mm	*S/V*	*D* mm
mean	*Leptidea juvernica*	0.81	0.76	1.07	0.96
	*Leptidea sinapis*	0.63	0.84	0.75	0.58
	both species	0.73	0.79	0.93	0.85
standard deviation	*Leptidea juvernica*	0.10	0.07	0.15	0.16
	*Leptidea sinapis*	0.07	0.07	0.07	0.03
	both species	0.13	0.08	0.20	0.22
T-criterion for differentiation between the species		5.60	2.84	7.49	9.42
significance		P < 0.001	P < 0.01	P < 0.001	P < 0.001

**Table 5.** CAPS-analysis data, the lengths of the saccus (*S*), valve (*V*) and their ratio (*S*/*V*) in males of *Leptidea sinapis* and *Leptidea juvernica*.

Specimen	CAPS-analysis data (gene/restriction endonuclease)			Measurements		
	*COI*/ *Hpa*II	*ITS2*/ *Alu*I	*CAD* *Hind*III	*S* mm	*V* mm	ratio *S*/*V*
L1	s	s	s	0.60	0.88	0.69
L2	j	j	j	0.83	0.73	1.14
L3	j	j	j	0.80	0.78	1.03
L4	s	s	s	0.63	0.80	0.78
L5	j	j	j	0.78	0.70	1.11
L6	s	s	s	0.45	0.70	0.64
L7	s	s	s	0.60	0.88	0.69
L8	j	j	j	0.88	0.70	1.25
L9	j	j	j	0.75	0.70	1.07
L10	j	j	j	0.60	0.75	0.80
L11	j	j	j	0.73	0.78	0.94
L12	j	j	j	0.75	0.75	1.00
L13	j	j	j	0.83	0.75	1.10
L14	j	j	j	0.90	0.80	1.13
L15	s	s	s	0.70	0.88	0.80
L16	s	s	s	0.55	0.88	0.63
L17	s	s	s	0.68	0.95	0.71
L18	s	s	s	0.68	0.93	0.73
L19	j	j	j	0.65	0.73	0.90
L20	j	j	j	0.95	0.75	1.27
L21	j	j	j	0.83	0.65	1.27
L22	s	s	s	0.68	0.85	0.79
L23	s	s	s	0.63	0.78	0.81
L24	j	j	j	0.80	0.95	0.84
L25	s	s	s	0.70	0.83	0.85
L26	s	s	s	0.63	0.75	0.83
L27	j	j	j	0.88	0.88	1.00
L28	j	j	j	0.98	0.80	1.22

**Table 6.** CAPS-analysis data and the lengths of the ductus in females of *Leptidea sinapis* and *Leptidea juvernica*.

Specimen	CAPS-analysis data (gene/restriction endonuclease)			The length of ductus (mm)
	*COI*/ *Hpa*II	*ITS2*/ *Alu*I	*CAD*/ *Hind*III	
L29	j	j	j	1.00
L30	j	j	j	1.10
L31	j	j	j	0.83
L32	j	j	j	0.78
L33	j	j	j	0.95
L34	s	s	s	0.60
L35	j	j	j	1.00
L36	j	j	j	0.95
L37	s	s	s	0.60
L38	j	j	j	0.88
L39	j	j	j	0.85
L40	j	j	j	1.23
L41	j	j	j	0.75
L42	j	j	j	1.25
L43	s	s	s	0.55
L44	s	s	s	0.55
L45	j	j	j	0.90
L46	s	s	s	0.60

**Table 7.** Additional characters: the saccus curvature, general size, hind wing underside suffusion and fore wing apex shape, classified to arbitrary classes, in the studied male specimens of *Leptidea sinapis* and *Leptidea juvernica*. Character states: saccus: 0 – straight, 1 – S-like curved; general size: 0 – large, 1 – small; hind wing underside suffusion below vein M3: 0 – with well-expressed lighter spots between veins, 1 – rather even, with very scarcely or not expressed lighter spots; fore wing apex: 0 – broadly rounded; 1 – more attenuated and acute. The typical *Leptidea sinapis* phenotype corresponds to the character states 0000, the typical *Leptidea juvernica* to 1111.

Specimen	Molecular identification	Saccus curvature	General size	Hind wing underside suffusion	Fore wing apex
L1	s	1	0	0	0
L2	j	1	1	1	1
L3	j	1	1	1	1
L4	s	1	0	1	1
L5	j	1	1	1	1
L6	s	0	0	0	0
L7	s	0	1	1	1
L8	j	1	1	1	0
L9	j	1	1	1	0
L10	j	1	0	1	1
L11	j	1	1	1	1
L12	j	1	1	1	0
L13	j	1	1	1	1
L14	j	1	1	1	1
L15	s	1	0	0	1
L16	s	1	1	0	1
L17	s	0	1	0	0
L18	s	0	0	0	0
L19	j	1	1	1	1
L20	j	1	1	1	1
L21	j	1	1	1	0
L22	s	1	0	0	0
L23	s	0	0	0	0
L24	j	1	1	1	1
L25	s	0	0	0	0
L26	s	1	1	1	0
L27	j	0	0	1	0
L28	j	1	0	1	0

**Figure 5.**
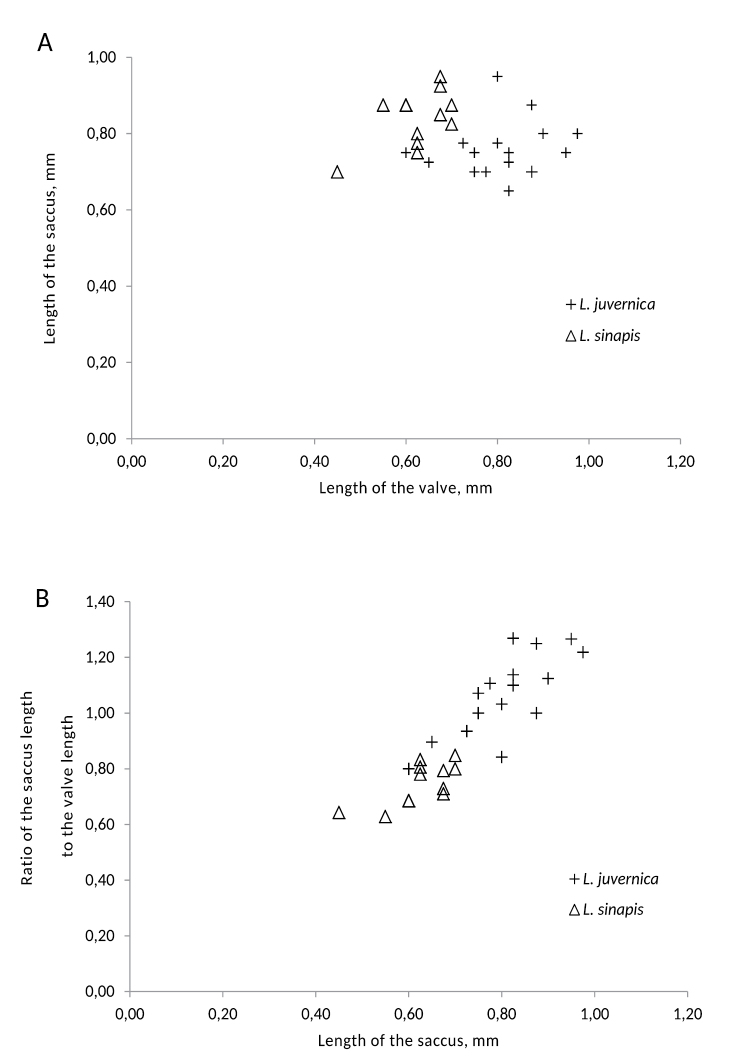
The saccus and valve lengths of the *Leptidea sinapis* and *Leptidea juvernica*. **A** Plot of the saccus length against the valve length of *Leptidea sinapis* and *Leptidea juvernica*, as identified by molecular markers **B** Plot of the ratio of the saccus length to the valve length against the saccus length for the same sample.

Two classes of spring brood females of the *s*- and *j*-groups with respect to the ductus bursae length were concordant with the CAPS data. The mean ductus length was significantly (p<.001) inferior in the s-group, and the length distributions of these groups did not overlap (Tables 4 and 6). In males, the difference between groups in the mean lengths of the saccus and valve were significant as well, with p<.001 and p<.01, respectively (Table 4). At the same time, the distributions of both the saccus and valve lengths of the *s*- and *j*-groups overlapped (Fig. 5).

The saccus curvature did not appear as a reliable differentiating character as well, since its mean square contingency coefficient (φ coefficient) value with the CAPS data was rather small (φ = 0.50, p<.001).

The size, coloration of the hind wing underside and the shape of apex of the fore wing were also found to associate with the molecular groups *j* and *s* but again with small values of the φ coefficient: φ = 0.49 for the size, φ = 0.73 for the hind wing coloration p<0.001; φ = 0.29 for the fore wing apex p<0.05.

It may be concluded that neither the genital structure lengths, nor the saccus curvature, nor the general size, nor the wing coloration allow reliable identification of males of the s- and j-groups.

## Discussion

### Genetic integrity of the species *Leptidea sinapis* and *Leptidea juvernica* in the studied location

The observed differences in the studied *COI* fragment of *Leptidea sinapis* and *Leptidea juvernica* are substantial. They are illustrated by the averaged and minimum p-distances provided in Table 3 (both values being very close to each other). This result well agrees with the earlier published data (Dincă et al. 2011, 2013, Lukhtanov et al. 2011). Full concordance of alleles of two unlinked nuclear genes and mitochondrial genes unequivocally supports the existence of integrated “molecular species” which can be identified based on diagnostic nucleotide substitutions in either of the mentioned genes, by CAPS-analysis or direct sequencing. Since any interspecies cross would bring about discordance of these markers, that we did not detect, the gene flow between these ‘molecular species’ is either absent or very limited. Thus, our ‘molecular species’ are at the same time valid biological species according to the Mayerian species concept. They are to be identified as taxonomical species *Leptidea sinapis* and *Leptidea juvernica*, according to the predominating morphological characters used to be considered diagnostic for species bearing these names. At the same time, we claim that these morphological characters are not satisfactory for species identification, since opposite variants of each of them are still present in each of the two species.

### Insufficiency of morphological and colorational characters for identification of the species *Leptidea sinapis* and *Leptidea juvernica*

In some studies the task of quick and still reliable identification of species of the *Leptidea sinapis* complex by application of a morphometric approach was achieved with a 100% efficiency (Fumi 2008, Sachanowicz 2013). In other cases, overlapping of morphometric characters was observed so that some specimens could not be unequivocally identified (Hauser 1997, Kudrna 2001, Verovnik and Glogovčan 2007). This could result from either insufficient genetic isolation of species or a greater intraspecific variation, e.g. driven by ecological factors (Bolshakov et al. 2013, Tsvetkov 2007, Fumi 2008) or from differences between the spring and summer brood (Sachanowicz 2013). Fumi (2008) also noted the potential effect of choosing poor diagnostic characters and measurement errors.

According to the discriminant criterion suggested by Fumi (2008) for females of *Leptidea sinapis* and *Leptidea reali*, the critical value for the ductus bursae length was 0.79 mm. According to our data, the hiatus of this character between females of *Leptidea sinapis* and *Leptidea juvernica* is at the interval of 0.60–0.75 mm.

The length of the saccus and valve in our case appeared insufficient for a complete discrimination of *Leptidea sinapis* and *Leptidea juvernica*. The ratio of these values, which allowed 98% discrimination of males of *Leptidea sinapis* and *Leptidea reali* (Fumi 2008), in our case also had better resolution but did not yet display a hiatus between the species (Fig. 5). Ideally, the way of discriminating should be unequivocal and not depend on geographical, ecological or seasonal circumstances. Fumi (2008) achieved 100% discrimination of males of *Leptidea sinapis* and *Leptidea reali* through simultaneous analysis of four genitalic characters: the lengths of the aedeagus, saccus, valve and uncus, while adding further characters to the multivariate analysis did not further contribute to resolution. However, discriminative analysis of several morphometric characters of the genitalia is laborious and hence impractical for routine identification of specimens. At present, molecular analysis involving either of our CAPS-markers is an easier means of identification a specimen than multivariate analysis of the genitalia morphology. Furthermore, the differentiation of *Leptidea reali* and *Leptidea juvernica* is still beyond the morphological approach and until now these species can be distinguished only by molecular markers and/or karyotype (Dincă et al. 2011, 2013).

Other external characters, such as the general size and coloration of the hind wing underside, recognised by a naked eye, are unreliable and allow only a first approach to species identification in the field (Ivonin et al. 2009), as follows from the low values of the φ coefficient for association of these characters with molecular markers. This may result from the conventional nature of the character grades or from the great variability for these characters, maybe as a remnant of introgression between species in the past. Note, however, that no males of *Leptidea juvernica* (identified by molecular markers) were scored as ‘0’ as to the hind wing underside suffusion (that is with well-expressed lighter spots below vein M3), the variant found in the majority of males of *Leptidea sinapis* (Table 7). This somewhat agrees with the data by Ivonin et al (2009) who did not find external characters of *Leptidea sinapis* among the males of *Leptidea juvernica* identified by the genitalia.

Anyway, we conclude that molecular and karyological characters are so far the only reliable means of identification of *Leptidea sinapis* and *Leptidea juvernica*, and the molecular ones are much easier methodically.

### Overlapping lengths of genital structures of *Leptidea sinapis* and *Leptidea juvernica*

Divergence and fixation of alleles of genes responsible for reproductive isolation are sufficient for speciation (Wu 2001). If genitalic differences contribute to reproductive isolation, genes governing the genital structures are to occur among them quite often. Differences in the genitalia size and structure result from the realization of the ontogenetic program. Since *Leptidea sinapis* and *Leptidea juvernica* are closely related species, they should have the same set of orthologous genes with a similar system of expression regulation in ontogenesis. A new allele in one of such ‘genital’ genes which once occurred in a small population can soon be fixed by gene drift, giving rise to a nascent genetically isolated species. Developmental genes often have pleiotropic effects and their mutation can bring about changes in a complex of morphological characters. In particular, the same gene may affect the length of both male and female genitalia through an effect on the size of the anlagen of genital organs in early embryogenesis in both sexes. Hence, the differences in the male and female genitalia between *Leptidea sinapis* and *Leptidea juvernica* may be determined by the same single major gene but also be influenced by small effects of an unknown number of other genetic and/or environmental factors. This seems to be common in *Leptidea*: thus, *Leptidea lactea* Lorkovic, 1950 was isolated from *Leptidea morsei* because of an observed bimodal distribution of the genitalia length in united samples (Lorcovic 1950).

The overlap of distribution of the length of the male genital structures may be interpreted through presence in both species of both ‘long’ and ‘short’ alleles of the hypothetical gene responsible for differences between *Leptidea sinapis* and *Leptidea juvernica*, although with oppositely biased frequencies. This could result from:

- inheritance of both alleles from the common ancestor,
- introgression between species, and
- de novo mutational re-appearance of ‘long’ and/or ‘short’ alleles.

Introgression is a common phenomenon for sympatric closely related butterfly species. According to an estimation by Mallet (2005), about 16% of 440 European butterfly species can hybridise with at least one other species in natural conditions. Most of such hybrids, especially females, suffer from lowered fertility or complete sterility in F1. However, some interspecific hybrids are still able of backcrossing with one of the parental species, which can lead to gene flow in hybrid zones (Mavárez et al. 2006, Descimon and Mallet 2009).

Specimens from Novosibirsk Province with intermediate state of diagnostic external characters or, more frequently, with discordant combination of characters of *Leptidea sinapis* and *Leptidea juvernica* were supposed to be interspecies hybrids or products of their backcrosses (Kosterin et al. 2007, Ivonin et al. 2009). In particular, Ivonin et al. (2009) claimed that among the spring brood males with the genitalia of *Leptidea sinapis*, there were specimens with the external characters of *Leptidea juvernica* (a dark, evenly suffused hind wing underside below vein M3, a processed fore wing apex). Oppositely, the males with *Leptidea juvernica* genitalia were homogenous for these characters. Our data do not support the latter claim, for the coloration of hind wing underside and the fore wing apex shape varied strongly in males of both *Leptidea sinapis* and *Leptidea juvernica*.

An attempt to reveal hybridization between *Leptidea sinapis* and *Leptidea reali* in the Pyrenees using 16 allozyme loci was unsuccessful (Martin et al. 2003). Verovnik and Glogovčan (2007) suspected a possible hybridisation between *Leptidea sinapis* and *Leptidea juvernica* in Slovenia. They revealed some unusual specimens which either had the saccus of an intermediate length or had a long saccus but short aedeagus. These specimens referred to *Leptidea sinapis* according to molecular markers. The same authors revealed two specimens morphologically corresponding to *Leptidea juvernica* but belonging to *Leptidea sinapis* according to the *COI* sequence. However, their RAPD analysis revealed fragments specific both to *Leptidea sinapis* and *Leptidea juvernica*, thus suggesting the hybrid nature of those specimens. The most recent large scale study did not detect signs of introgression among the three species of the *Leptidea sinapis* complex (Dincă et al. 2013). On the contrary, the existence of biochemical and behavioural prezygotic barriers among them were demonstrated (Friberg et al. 2008b, Dincă et al. 2013).

Inheritance of ‘long’ and ‘short’ alleles in the common ancestor of *Leptidea sinapis* and *Leptidea juvernica* is also a plausible interpretation. These alleles could be involved in genetic isolation of the nascent species by forming a reproductive barrier between them, but fixation of either allele in these species may not have taken place. The initial genital prezygotic barrier could later be strengthened by adding biochemical and behavioral barriers. They would lower significance of the primary genital barrier and somewhat release selection for the ‘long’ versus ‘short’ alleles and vice versa, allowing their frequency to drift.

At this stage of our knowledge, the third scenario of arising ‘long’ and/or ‘short’ allele(s) cannot be excluded as well.

### Variability of common markers versus conservation of histone H1 gene

In contrast to core histones, histone H1 is a very variable protein (Berdnikov et al. 1993, Happel and Doenecke 2009). For this reason its gene served well for reconstructing phylogeny of the genus *Pisum* L. (peas) both at inter- and intraspecies level (Zaytseva et al. 2012, 2015). In spite of its variability elsewhere, the *H1* gene appeared identical in *Leptidea sinapis* and *Leptidea juvernica*. The *H1* gene variation revealed in the four studied species of *Leptidea* turned out to be five times lower than that of *COI*, differing from our data obtained for three species of *Oreta* Walker, 1955 (Drepanidae) (Solovyev et al. 2015), where the substitution rate in *H1* appeared to be only twice less than that in *COI*.

### Three *wsp* alleles in four *Leptidea* species

All the four studied *Leptidea* species were found to be infected with *Wolbachia*, with prevalence of infected specimens of 91.8% in *Leptidea sinapis* + *Leptidea juvernica* and 100% in *Leptidea amurensis* and *Leptidea morsei* (Table 1). We cannot exclude the possibility that all individuals in the studied populations are infected. We removed the abdomen and hence isolated DNA from somatic tissues only, while the *Wolbachia* presence can be limited to reproductive tissues (Dobson et al. 1999). The high level of infection probably indicates at functional effect of *Wolbachia* on the host, ranging from mutualism (increase of the host fitness) to a reproductive parasitism (cytoplasmic incompatibility, feminization, parthenogenesis, male-killing) (Werren 1997; Werren et al. 2008). We can exclude feminisation, male-killing or parthenogenesis that would result in biased sex ratio, which was not observed (Table 1). Special experimental studies would be necessary to investigate the reason of the high infection level in these four *Leptidea* species.

*Wolbachia* infection is vertically transmitted through host generations via maternal cytoplasm. Therefore the phylogeny of *Wolbachia* could be expected to be concordant with the phylogeny of its hosts. However, *Wolbachia* can as well be transmitted horizontally between related species through introgression and between unrelated species by unknown agents. In addition, *Wolbachia* strains as well as their particular genes such as *wsp* may result from recombination between different strains.

We found three *Wolbachia* strains in *Leptidea* according to the *wsp* gene sequences. Three species, *Leptidea amurensis*, *Leptidea sinapis* and *Leptidea juvernica*, were found to possess allele *wsp*- 10 which is widespread in insects. According to our counts at the http://pubmlst.org, it was so far registered in 27 species of Lepidoptera from different families (Pyralidae, Hesperiidae, Papilionidae, Pieridae, Nymphalidae, Lycaenidae) and, and also in *Culex pipiens* Linnaeus, 1758 from Diptera. The second allele *wsp-687* was found in *Leptidea sinapis* for the first time. It differs from wsp-10 in only one nucleotide substitution. The third *wsp*-688 allele was also found for the first time, in *Leptidea morsei*. This new allele has a unique hypervariable region 2, *HVR2-267*, while other hypervariable regions *HVR1-2*, *HVR3-2*, *HVR-23* were found elsewhere (Baldo et al. 2005, 2010; Baldo and Werrein 2007). Nevertheless the mentioned combination of the hypervariable regions has not been so far recorded, thus the allele is a product of recombination of strains representing different evolutionary lines of *Wolbachia*.

The pattern of *Wolbachia* variants in the studied *Leptidea* species is discordant to the host phylogeny. Variation of *wsp* sequences in *Leptidea sinapis*, *Leptidea juvernica* and *Leptidea amurensis* is extremely low, viz. *wsp-10* allele is common for these species and a closely related *wsp-687* allele is also found in *Leptidea sinapis*, whereas *Leptidea morsei* possesses a highly divergent *wsp*-688 allele.

The *wsp-10* allele could hardly be inherited from the common ancestor of the three species, taking into account a considerable degree of variation accumulated by the host *Leptidea* genes, both nuclear and mitochondrial (Dincă et al. 2011, 2013 and this paper). Note that supposition (i) contradicts also the phylogenetic relationships of the species involved (Dincă et al. 2011) as follows: the branch *Leptidea morsei* + *Leptidea amurensis* is opposed to the branch containing *Leptidea sinapis* and *Leptidea juvernica*. The low *wsp* variation in three species may have two explanations:

- the same strain of *Wolbachia* could have spread across the three species by interspecies crosses;
- The same *wsp* allele could have spread across the three species via horizontal transfer of *Wolbachia*.

We exclude option (i) since we failed to trace such crosses by other molecular means. Explanation (ii), that is independent infection by the same *Wolbachia* strain, is most probable because of a high frequency of *wsp-10* in butterflies. *Leptidea morsei* was no doubt independently infected by an unusual *Wolbachia* strain with *wsp*-688, however, more data on *Leptidea morsei* is necessary to consider the evolutionary history of its *Wolbachia*.

## Conclusions

*Leptidea amurensis*, *Leptidea morsei*, *Leptidea sinapis* and *Leptidea juvernica* coexist in the same locality in West Siberia without detectable introgression. Each of the molecular characters *COI*, *CAD* and *ITS2* markers, as well as the length of the female ductus bursae, allow a reliable identification of *Leptidea sinapis* and *Leptidea juvernica*. The length of the saccus related to that of the valva as the most easily assessed male genitalic character, as well as the characters of wing pattern and shape in males, are unreliable for identification of these two species. An overwhelming majority of *Leptidea* individuals are infected with *Wolbachia*. Three alleles of the *Wolbachia* gene *wsp* were recorded (two of them for the first time), that of *Leptidea morsei* being highly divergent from the allele found in *Leptidea amurensis*, *Leptidea juvernica* and *Leptidea sinapis* (this species contains a very similar third allele), which is discordant with the presumed phylogeny of the host.

## References

[B1] Baldo L Desjardins CA Russel JA Stahlhut JH Werren JH (2010) Accelerated microevolution in an outer membrane protein (OMP) of the intracellular bacteria *Wolbachia*. BMC evolutionary biology 10(1): . doi: 10.1186/1471-2148-10-48 10.1186/1471-2148-10-48 PMC2843615 20163713

[B2] Baldo L Lo N Werren JH (2005) Mosaic nature of the *Wolbachia* surface protein. Journal of Bacteriology 187(15): 5406–5418. doi: 10.1128/JB.187.15.5406-5418.2005 16030235 10.1128/JB.187.15.5406-5418.2005 PMC1196003

[B3] Baldo L Werren JH (2007) Revisiting *Wolbachia* supergroup typing based on WSP: spurious lineages and discordance with MLST. Current Microbiology 55(1): 81–87. doi: 10.1007/s00284-007-0055-8 17551786 10.1007/s00284-007-0055-8

[B4] Berdnikov VA Rozov SM Temnykh SV Gorel’ FL Kosterin OE (1993) Adaptive nature of interspecies variation of Histone H1 in insects. Journal of Molecular Evolution 36(5): 497–507. doi: 10.1007/BF02406725

[B5] Bogdanova VS Galieva ER Kosterin OE (2009) Genetic analysis of nuclear-cytoplasmic incompatibility in pea associated with cytoplasm of an accession of wild subspecies Pisum sativum subsp. elatius (Bieb.) Schmahl. Theoretical and Applied Genetics 118(4): 801–809. doi: 10.1007/s00122-008-0940-y 19099285 10.1007/s00122-008-0940-y

[B6] Bolshakov LV (2005) Variability and problems of intraspecific systematics of *Leptidea reali* Reissinger, 1989 (Lepidoptera: Pieridae) in European Russia and neighboring regions (with discussion of synonymy and new findings of some related species). Eversmannia. Entomological Research in Russia and Adjacent Regions 1: 4–12. [In Russian] http://www.eversmannia.entomology.ru/eversmannia_01_04.pdf

[B7] Bolshakov LV Ruchin AB Kurmaeva DK (2013) Fauna and variability of the genus *Leptidea* Billberg, 1820 (Lepidoptera, Pieridae) in the Republic of Mordovia, Russia. Euroasian Entomological Journal 12(1): 87–92. [In Russian]

[B8] Braig HR Zhou W Dobson SL O’Neill SL (1998) Cloning and characterization of a gene encoding the major surface protein of the bacterial endosymbiont *Wolbachia pipientis*. Journal of Bacteriology 180(9): 2373–2378. 9573188 10.1128/jb.180.9.2373-2378.1998 PMC107178

[B9] Descimon H Mallet J (2009) Bad species. In: Settele J Konvicka M Shreeve T Van Dyck H (Eds) Ecology of Butterflies in Europe. Cambridge, 219–249.

[B10] Dincă V Lukhtanov VA Talavera G Vila R (2011) Unexpected layers of cryptic diversity in wood white *Leptidea* butterflies. Nature communications 2: 324. doi: 10.1038/ncomms1329 10.1038/ncomms1329 21610727

[B11] Dincă V Wiklund C Lukhtanov VA Kodandaramaiah U Norén K Dapporto L Friberg M (2013) Reproductive isolation and patterns of genetic differentiation in a cryptic butterfly species complex. Journal of Evolutionary Biology 26(10): 2095–2106. doi: 10.1111/jeb.12211 23909947 10.1111/jeb.12211 PMC4413813

[B12] Dobson SL Bourtzis K Braig HR Jones BF Zhou W Rousset F O’Neill SL (1999) *Wolbachia* infections are distributed throughout insect somatic and germ line tissues. Insect Biochemistry and Molecular Biology 29(2): 153–160. doi: 10.1016/S0965-1748(98)00119-2 10196738 10.1016/s0965-1748(98)00119-2

[B13] Friberg M Bergman M Kullberg J Wahlberg N Wiklund C (2008a) Niche separation in space and time between two sympatric sister species — a case of ecological pleiotropy. Evolutionary Ecology 22(1): 1–18. doi: 10.1007/s10682-007-9155-y

[B14] Friberg M Vongvanich N Borg-Karlson AK Kemp DJ Merilaita S Wiklund C (2008b) Female mate choice determines reproductive isolation between sympatric butterflies. Behavioral Ecology and Sociobiology 62(6): 873–886. doi: 10.1007/s00265-007-0511-2

[B15] Friberg M Wiklund C (2009) Host plant preference and performance of the sibling species of butterflies *Leptidea sinapis* and *Leptidea reali*: a test of the trade-off hypothesis for food specialization. Oecologia 159(1): 127–137. doi: 10.1007/s00442-008-1206-8 19002503 10.1007/s00442-008-1206-8

[B16] Folmer O Black M Hoeh W Lutz R Vrijenhoek R (1994) DNA primers for amplification of mitochondrial cytochrome c oxidase subunit I from diverse metazoan invertebrates. Molecular Marine Biology and Biotechnology 3(5): 294–299. http://www.mbari.org/staff/vrijen/PDFS/Folmer_94MMBB.pdf 7881515

[B17] Fumi M (2008) Distinguishing between *Leptidea sinapis* and *L. reali* (Lepidoptera: Pieridae) using a morphometric approach: impact of measurement error on the discriminative characters. Zootaxa 1819: 40–54. http://www.mapress.com/zootaxa/2008/f/z01819p054f.pdf

[B18] Happel N Doenecke D (2009) Histone H1 and its isoforms: contribution to chromatin structure and function. Gene 431(1–12): 1–12. doi: 10.1016/j.gene.2008.11.003 19059319 10.1016/j.gene.2008.11.003

[B19] Hauser E (1997) *Leptidea sinapis* (Linnaeus 1758) und *Leptidea reali* Reissinger 1989: zwei verschiedene Arten? (Lepidoptera, Pieridae). Beiträge zur Naturkunde Oberösterreichs 5: 65–75.

[B20] Ivonin VV Kosterin OE Nikolaev SL (2009) Butterflies (Lepidoptera, Diurna) of Novosibirsk Province, Russia. 1. Hesperiidae, Papiliondae, Pieridae. Euroasian Entomological Journal 8(1): 85–104.

[B21] Konieczny A Ausubel FM (1993) A procedure for mapping *Arabidopsis* mutations using co-dominant ecotype-specific PCR-based markers. The Plant Journal 4(2): 403–410. doi: 10.1046/j.1365-313X.1993.04020403.x 8106085 10.1046/j.1365-313x.1993.04020403.x

[B22] Kosterin OE Sergeev MG Dubatolov VV (2007) Butterflies (Lepidoptera, Diurna). In: Zhimulev IF (Ed.) Nature of Academy Town: 50 Years After. Novosibirsk, 105–133. [In Russian]

[B23] Kudrna O (2001) Miscellaneous notes on the taxonomy of four European butterflies (Lepidoptera: Rhopalocera). Entomologist’s Gazette 52: 253–261.

[B24] Mallet J (2005) Hybridization as an invasion of the genome. Trends in Ecology and Evolution 20(5): 229–237. doi: 10.1016/j.tree.2005.02.010 16701374 10.1016/j.tree.2005.02.010

[B25] Martin JF Gilles A Descimon H (2003) Species concepts and sibling species: the case of *Leptidea sinapis* and *Leptidea reali*. In: Boggs CL Watt WB Ehrlich P (Eds) Butterflies. Ecology and Evolution Taking Flight. Chicago, 459–476.

[B26] Mavárez J Salazar CA Bermingham E Salcedo C Jiggins CD Linares M (2006) Speciation by hybridization in *Heliconius* butterflies. Nature 441: 868–871. doi: 10.1038/nature04738 16778888 10.1038/nature04738

[B27] Lorkovic Z (1950) Neue ostasiatische Arten und Rassen der Gattung *Leptidea* nebst Nomenklaturberichtigungen. Glasnik Biloske Sekcije Periodicum Biologorum (Zagreb) 2/3: 57–76. http://www.euroleps.ch/seiten/s_lit.php?lit=lorkovic1950&art=pier_lactea&ressort=OD

[B28] Lukhtanov VA Dincă V Talavera G Vila R (2011) Unprecedented within-species chromosome number cline in the Wood White butterfly *Leptidea sinapis* and its significance for karyotype evolution and speciation. BMC evolutionary biology 11(1): . doi: 10.1186/1471-2148-11-109 10.1186/1471-2148-11-109 PMC3113740 21507222

[B29] Réal P (1988) Lépidoptères nouveaux principalement jurassiens. Mémoires du Comité de Liaison pour les Recherches Ecofaunistiques dans le Jura 4: 1–28.

[B30] Reissinger E (1989 [1990]) Checkliste Pieridae Duponchel, 1835, der Westpalaearktis (Europa, Nordwestafrika, Kaukasus, Kleinasien). Atalanta 20: 149–185.

[B31] Sachanowicz K (2013) Separation possibilities and genital measurement variations in two cryptic species of European pierid butterﬂies, *Leptidea juvernica* Williams, 1946 and *L. sinapis* (Linnaeus, 1758). Zoology 116(4): 215–223. doi: 10.1016/j.zool.2012.12.002 23827501 10.1016/j.zool.2012.12.002

[B32] Solovyev VI Bogdanova VS Dubatolov VV Kosterin OE (2015) Range of a Palearctic uraniid moth *Eversmannia exornata* (Lepidoptera: Uraniidae: Epipleminae) was split in the Holocene, as evaluated using histone H1 and COI genes with reference to the Beringian disjunction in the genus *Oreta* (Lepidoptera: Drepanidae). Organisms Diversity and Evolution 15(2): 285–300. doi: 10.1007/s13127-014-0195-1

[B33] Tamura K Peterson D Peterson N Stecher G Nei M Kumar S (2011) MEGA5: molecular evolutionary genetics analysis using maximum likelihood, evolutionary distance, and maximum parsimony methods. Molecular Biology and Evolution 28(10): 2731–2739. doi: 10.1093/molbev/msr121 21546353 10.1093/molbev/msr121 PMC3203626

[B34] Tsvetkov EV (2007) On *Leptidea reali* Reissinger, 1989 (Lepidoptera: Pieridae) genitalia structure variability in Leningrad and Voronezh Areas. Eversmannia 11–12: 19–23. http://eversmannia.entomology.ru/eversmannia_11-12_19.pdf

[B35] Van Meer MM Witteveldt J Stouthamer R (1999) Phylogeny of the arthropod endosymbiont *Wolbachia* based on the *wsp* gene. Insect Molecular Biology 8(3): 399–408. doi: 10.1046/j.1365-2583.1999.83129.x 10469257 10.1046/j.1365-2583.1999.83129.x

[B36] Verovnik R Glogovčan P (2007) Morphological and molecular evidence of a possible hybrid zone of *Leptidea sinapis* and *L. reali* (Lepidoptera: Pieridae). European Journal of Entomology 104(4): 667–674. http://www.eje.cz/pdfs/eje/2007/04/05.pdf

[B37] Werren JH (1997) Biology of *Wolbachia*. Annual Review of Entomology 42(1): 587–609. doi: 10.1146/annurev.ento.42.1.587 10.1146/annurev.ento.42.1.587 15012323

[B38] Werren JH Baldo L Clark ME (2008) *Wolbachia*: master manipulators of invertebrate biology. Nature Reviews Microbiology 6(10): 741–751. doi: 10.1038/nrmicro1969 18794912 10.1038/nrmicro1969

[B39] Wu C-I (2001) The genic view of the process of speciation. Journal of Evolutionary Biology 14(6): 851–865. doi: 10.1046/j.1420-9101.2001.00335.x

[B40] Zaytseva OO Bogdanova VS Kosterin OE (2012) Phylogenetic reconstruction at the species and intraspecies levels in the genus *Pisum* (L.) (peas) using a histone H1 gene. Gene 504(2): 192–202. doi: 10.1016/j.gene.2012.05.026 22613846 10.1016/j.gene.2012.05.026

[B41] Zaytseva OO Gunbin KV Mglinets AV Kosterin OE (2015) Divergence and population traits in evolution of the genus *Pisum* L. as reconstructed using genes of two histone H1 subtypes showing different phylogenetic resolution. Gene 556(2): 235–244. doi: 10.1016/j.gene.2014.11.062 25476028 10.1016/j.gene.2014.11.062

[B42] Zhou W Rousset F O’Neil S (1998) Phylogeny and PCR-based classification of *Wolbachia* strains using *wsp* gene sequences. Proceedings of Biological Sciences 265(1395): 509–15. doi: 10.1098/rspb.1998.0324 10.1098/rspb.1998.0324 PMC1688917 9569669

